# Family physicians' involvement in palliative cancer care

**DOI:** 10.1002/cam4.5371

**Published:** 2022-10-20

**Authors:** Christine C. Moon, Kenneth Mah, Ashley Pope, Nadia Swami, Breffni Hannon, Jenny Lau, Ernie Mak, Ahmed al‐Awamer, Subrata Banerjee, Laura A. Dawson, Amna Husain, Gary Rodin, Lisa W. Le, Camilla Zimmermann

**Affiliations:** ^1^ Department of Supportive Care, Princess Margaret Cancer Centre University Health Network Toronto Ontario Canada; ^2^ Department of Medicine University of Toronto Toronto Ontario Canada; ^3^ Department of Family and Community Medicine University of Toronto Toronto Ontario Canada; ^4^ Department of Radiation Oncology University of Toronto, Radiation Medicine Program, Princess Margaret Cancer Centre Toronto Ontario Canada; ^5^ Temmy Latner Centre for Palliative Care Lunenfeld Tanenbaum Research Institute, Sinai Health Division of Palliative Care University of Toronto Toronto Ontario Canada; ^6^ Department of Psychiatry University of Toronto Toronto Ontario Canada; ^7^ Global Institute of Psychosocial, Palliative and End‐of‐Life Care (GIPPEC) University of Toronto and Princess Margaret Cancer Centre Toronto Ontario Canada; ^8^ Department of Biostatistics, Princess Margaret Cancer Centre University Health Network Toronto Ontario Canada; ^9^ Princess Margaret Research Institute, Princess Margaret Cancer Centre University Health Network Toronto Ontario Canada

**Keywords:** cancer, cross‐sectional survey, family physicians, palliative care, satisfaction with care

## Abstract

**Background:**

Family physicians' (FPs) long‐term relationships with their oncology patients position them ideally to provide primary palliative care, yet their involvement is variable. We examined perceptions of FP involvement among outpatients receiving palliative care at a cancer center and identified factors associated with this involvement.

**Methods:**

Patients with advanced cancer attending an oncology palliative care clinic (OPCC) completed a 25‐item survey. Eligible patients had seen an FP within 5 years. Binary multivariable logistic regression analyses were conducted to identify factors associated with (1) having seen an FP for palliative care within 6 months, and (2) having a scheduled/planned FP appointment.

**Results:**

Of 258 patients, 35.2% (89/253) had seen an FP for palliative care within the preceding 6 months, and 51.2% (130/254) had a scheduled/planned FP appointment. Shorter travel time to FP (odds ratio [OR] = 0.67, 95% confidence interval [CI] = 0.48–0.93, *p* = 0.02), the FP having a 24‐h support service (OR = 1.96, 95% CI = 1.02–3.76, *p* = 0.04), and a positive perception of FP's care (OR = 1.05, 95% CI = 1.01–1.09, *p* = 0.01) were associated with having seen the FP for palliative care. English as a first language (OR = 2.90, 95% CI = 1.04–8.11, *p* = 0.04) and greater ease contacting FP after hours (OR = 1.33, 95% CI = 1.08–1.64, *p* = 0.008) were positively associated, and female sex of patient (OR = 0.51, 95% CI = 0.30–0.87, *p* = 0.01) and travel time to FP (OR = 0.66, 95% CI = 0.47–0.93, *p* = 0.02) negatively associated with having a scheduled/planned FP appointment. Number of OPCC visits was not associated with either outcome.

**Conclusion:**

Most patients had not seen an FP for palliative care. Accessibility, availability, and equity are important factors to consider when planning interventions to encourage and facilitate access to FPs for palliative care.

## 1 INTRODUCTION

Early outpatient palliative care improves quality of life, symptom control, and satisfaction with care for patients with advanced cancer.^1^, ^2^, ^3^ While clinical trials of early palliative care have predominantly examined specialized palliative care interventions, it is increasingly recognized that a sustainable model requires both generalist and specialist palliative care.^4^ Family physicians (FPs) are ideally positioned to provide basic palliative care, with specialized palliative care physicians providing care in more complex situations.^4^, ^5^, ^6^, ^7^ FPs have long‐term relationships with patients and their families, which enable continuity of care and smooth transitions to end‐of‐life care and facilitate provision of psychosocial support and eventual bereavement care.^8^, ^9^, ^10^, ^11^, ^12^ However, receiving oncological care at a cancer center may supplant FPs' care provision.^13^ In addition, an increasing number of cancer centers have outpatient palliative care clinics, where patients receive specialized symptom control, advance care planning, and emotional support.^10^, ^14^ Although the intent of these clinics is to provide specialized palliative care in collaboration with FPs and oncologists,^15^ it is possible that longitudinal follow‐up in the outpatient palliative care clinic could compromise the receipt of palliative care by FPs.

Factors that may facilitate or impede FP involvement in palliative care for patients with advanced cancer can be conceptualized using Andersen's Behavioral Model of Health Services Use, which describes four main groups of factors determining access to and use of healthcare services.^16^, ^17^ *Predisposing factors* include patient‐related factors, such as demographic characteristics, that affect the use of healthcare services. *Enabling factors* are conditions, such as transportation and travel time, that facilitate or impede use of services. *Need factors* include conditions that reflect a requirement for medical treatment, such as the diagnosis of a medical illness. *Outcome factors* include patient‐reported outcomes, such as symptom control and satisfaction with care; these outcomes are influenced by the predisposing, enabling and need factors and, in turn, affect subsequent healthcare use.

Although the perspective of FPs on their role in providing cancer palliative care has been well described,^18^, ^19^, ^20^, ^21^, ^22^, ^23^, ^24^, ^25^ research on patients' perspectives of FP involvement in their palliative care consists mostly of small qualitative studies.^26^ The aims of the present study were to describe patient‐reported involvement of FPs in their palliative care and to identify factors associated with such involvement among patients with advanced cancer referred to an outpatient palliative care clinic.

## 2 METHODS

### 2.1 Participants

Participants were patients with cancer who were attending the Oncology Palliative Care Clinic (OPCC) of the Princess Margaret Cancer Centre, a large integrated cancer treatment, teaching, and research center in Toronto, Canada. The OPCC offers specialized palliative care services to outpatients with cancer, receiving approximately 1500 new referrals annually.^10^ To avoid selection bias, eligible patients were identified through screening of daily clinic patient lists for the OPCC. Inclusion criteria included attending the OPCC; age ≥18 years; ability to understand English sufficiently to provide informed consent and complete the study questionnaires; currently have, or previously had, an FP in the 5 years prior to recruitment; and physical and cognitive capacities to participate, according to their palliative care physician.

### 2.2 Materials

Participants' sociodemographic and medical characteristics were extracted from their medical charts using a chart review form developed for the study. A survey was developed to assess patients' perspectives of their FPs' involvement in their care. The survey comprised 25 multi‐component questions with a completion time of approximately 20 minutes. Two categorical survey items formed the two primary study outcomes: (1) having seen the FP for palliative care services (symptom control, advance care planning, and emotional care for cancer) in the last 6 months (versus ≥7 months or never); and (2) having scheduled or planning to schedule an appointment with the FP (versus not having scheduled and not planning to schedule an appointment). Two questions addressed from whom patients currently received and would prefer to receive various cancer and general medical services.

The remaining questions included items regarding predisposing factors (e.g. patient demographics), enabling factors (e.g. length of time with FP, travel time to FP's office, provision of home visits), and outcome factors (e.g. perception of medical care). For perceptions of medical care by the FP, seven survey items were rated from 0 (strongly disagree) to 4 (strongly agree): satisfaction with the FP's care; confidence in receiving the best care from the FP; feeling that the FP's role in their care was clear; feeling that the FP provided sufficient time to address their problems; feeling that the FP knew them as a person; receipt of prompt care from their FP; and availability of the FP for desired services. Given the mostly high intercorrelations among these seven items (Spearman's *ρ* = 0.46–0.87) and high internal consistency (Cronbach's *α* = 0.92), their ratings were summed to create a total score reflecting patients' perception of medical care for use in analyses. An additional item—feeling that it was easy to reach the FP or their team after hours—was included separately to reflect a specific, important aspect of palliative care and was rated on the same 0 to 4 scale.^27^, ^28^ This item demonstrated lower correlations with the seven perceptions of medical care items (Spearman's *ρ* = 0.19–0.39).

Additional outcome factors, which could also be conceptualized as need factors, were the Patient‐Reported Functional Status (PRFS) measure, a validated adaptation of the clinician‐rated ECOG^29^ and the Revised Edmonton Symptom Assessment System‐CS (ESASr‐CS) scale. The latter scale measures the severity of 11 common cancer symptoms: pain, tiredness, drowsiness, nausea, appetite, shortness of breath, depression, anxiety, well‐being, constipation, and sleep.^30^ Average severity of each symptom over the last 24 h was rated from 0 (best) to 10 (worst). A total summed score was calculated, as well as a physical subscale (pain, tiredness, drowsiness, nausea, appetite, shortness of breath, constipation, and sleep) and an emotional subscale (depression and anxiety). A chart review was conducted to abstract additional need factors including cancer diagnosis and number of previous visits to the OPCC.

### 2.3 Procedure

The study received approval from the University Health Network Research Ethics Board (REB #16‐5061‐CE). Participant recruitment in the OPCC was conducted between May 2016 and August 2018. Study staff approached the attending physicians or nurses to confirm eligible patients' physical and cognitive abilities to participate and then approached patients about participating in the study. After receiving information about the study, patients willing to participate provided written informed consent. They either completed the survey immediately or took it home to complete and return by mail using a provided addressed, stamped envelope.

### 2.4 Statistical methodology

The target sample size was 250 participants. Based on our clinical experience with the study population, we estimated that 25% of patients would have seen a FP for palliative care in the last 6 months; thus, with 250 patients, we would estimate that about 63 participants (250 × 25%) would have seen their family physician for palliative care in the last 6 months. Given the minimal number of events per predictor variable (EPV) of ≥10 that would result in valid regression coefficients,^31^ we would be able to accommodate five to six variables in a multivariate logistic regression analysis.

Descriptive statistics were used for patients' demographic and medical characteristics and the characteristics of their FP and their care services. Comparisons of groups within each outcome on patients' demographic and medical characteristics were conducted using chi‐square tests, Cochran‐Armitage trend test, and *t*‐tests. Univariable binary logistic regression was used to determine the relationship between positive perception of medical care and each outcome; this was done for the seven perception items individually and for the single score reflecting overall perception of the FP's medical care.

Binary logistic regression analyses were conducted to identify factors associated with the two outcomes. Candidate factors included the following variables from the survey, classified according to Andersen's model^16^, ^17^: (1) predisposing patient factors: age, sex, marital status, ethnic background, first language, and education level; (2) enabling factors: duration of relationship with the FP, travel time to FP's office, provision of home visits during or after office hours, provision of 24‐h telephone support, and ease of reaching the FP after hours; (3) need factors: time since diagnosis, current oncology treatment, and number of previous OPCC visits; and (4) outcome factors: symptom severity (ESASr‐CS total and physical and emotional subscales), perception of medical care total score, and PRFS rating. Factors that were associated with each outcome at *p* < 0.25 were entered into the respective stepwise selection procedures; factors with *p* < 0.05 were retained in the multivariable logistic regression model. Odds ratios (OR) and 95% confidence intervals (CI) for significant factors were reported. Analyses were performed on all available data and conducted using SPSS version 25 and SAS version 9.4. The significance level was set to 0.05.

## 3 RESULTS

### 3.1 Participant characteristics

A total of 832 patients were screened. Of these, 289 (34.7%) were ineligible (169 [20.3%] had a language barrier, 55 [6.6%] were too ill to approach, 23 [2.8%] had a cognitive deficit, 17 [2.0%] had no FP, and, for 25 [3.0%], clinicians asked that patients not be approached). The remaining 543 eligible patients (65.3%) were approached at least once. Of these, 280 (51.6%) declined participation (150 [27.6%] were not interested, 80 [14.7%] felt the study would take too much time or be burdensome, 44 [8.1%] were not feeling well, and 6 [1.1%] expressed dissatisfaction with their FP). The remaining 263 (48.4%) consented to the study, and 258 (47.5%) completed the survey.

Of those who responded, 89/253 (35.2%; five missing responses) reported having seen their FP for palliative care in the last 6 months, whereas 130/254 (51.2%; four missing responses) had scheduled or planned to schedule an appointment with their FP. Table 1 summarizes the demographic and medical characteristics of the 258 participants by each of these two outcomes. There were no significant differences in demographic or medical characteristics between those who had or had not seen their FP for palliative care within the last 6 months. Participants who had a scheduled or planned appointment with the FP were less likely to be female (*p* = 0.02) and more likely to speak English as a first language (*p* = 0.02) than those who did not have a scheduled or planned appointment.

**TABLE 1:** Patients' characteristics by previous and planned family physician visits

Characteristics	Total sample (*N* = 258), *n* (%)	Visited family physician for palliative care in last 6 months (*N* = 253), *n* (%)			Scheduled/plan to schedule appointment with family physician (*N* = 254), *n* (%)		
		Yes, *n* = 89	No, *n* = 164	*p*	Yes, *n* = 130	No, *n* = 124	*p*
Age, mean years (SD)	59.59 (12.2)	59.5 (11.6)	59.5 (12.5)	1.00	59.9 (12.5)	59.2 (12.0)	0.65
Sex				0.26			0.02
Female	143 (55.4)	45 (50.6)	95 (57.9)		62 (47.7)	78 (62.9)	
Male	115 (44.6)	44 (49.4)	69 (42.1)		68 (52.3)	46 (37.1)	
Marital status				0.87			0.87
Married/common‐law	161 (62.9)	56 (63.6)	102 (62.6)		82 (63.6)	77 (62.6)	
Other	95 (37.1)	32 (36.4)	61 (37.4)		47 (36.4)	46 (37.4)	
Ethnic background				0.87			0.06
European	177 (68.6)	61 (68.5)	114 (69.5)		97 (74.6)	79 (63.7)	
Other	81 (31.4)	28 (31.5)	50 (30.5)		33 (25.4)	45 (36.3)	
First language				0.90			0.02
English	235 (91.1)	81 (91.0)	150 (91.5)		124 (95.4)	108 (87.1)	
Other	23 (8.9)	8 (9.0)	14 (8.5)		6 (4.6)	16 (12.9)	
Education				0.48			0.61
≤High school	68 (26.4)	25 (28.1)	42 (25.6)		35 (26.9)	32 (25.8)	
Trades/college	58 (22.5)	23 (25.8)	34 (20.7)		32 (24.6)	25 (20.2)	
University	132 (51.2)	41 (46.1)	88 (53.7)		63 (48.5)	67 (54.0)	
Cancer site				0.62			0.41
Breast	31 (12.0)	10 (11.2)	21 (12.8)		15 (11.5)	16 (12.9)	
Gastrointestinal	66 (25.6)	26 (29.2)	39 (23.8)		32 (24.6)	33 (26.6)	
Genitourinary	37 (14.3)	12 (13.5)	24 (14.6)		24 (18.5)	13 (10.5)	
Gynecologic	28 (10.9)	12 (13.5)	16 (9.8)		10 (7.7)	18 (14.5)	
Head and neck	21 (8.1)	8 (9.0)	12 (7.3)		10 (7.7)	10 (8.1)	
Lung	37 (14.3)	13 (14.6)	24 (14.6)		21 (16.2)	16 (12.9)	
Other	38 (14.7)	8 (9.0)	28 (17.1)		18 (13.8)	18 (14.5)	
Time since diagnosis, mean years (SD)	4.06 (5.0)	3.73 (4.3)	4.33 (5.4)	0.37	4.10 (4.9)	4.08 (5.2)	0.98
Active cancer treatment				0.79			0.25
Yes	162 (62.8)	56 (62.9)	106 (64.6)		78 (60.0)	83 (66.9)	
No	96 (37.2)	33 (37.1)	58 (35.4)		52 (40.0)	41 (33.1)	
ESASr‐CS symptoms, mean (SD)							
Total score	30.90 (17.37)	31.81 (17.54)	30.73 (17.49)	0.54	30.17 (18.07)	31.40 (16.72)	0.58
Physical subscale	22.37 (12.94)	23.02 (12.69)	21.98 (13.20)	0.55	21.70 (13.21)	22.90 (12.71)	0.47
Emotional subscale	4.73 (4.62)	4.81 (4.83)	4.69 (4.56)	0.85	4.90 (4.86)	4.45 (4.39)	0.45
PRFS				0.41			0.72
0	19 (7.9)	2 (0.4)	17 (11.2)		8 (6.6)	10 (8.7)	
1	134 (55.8)	50 (60.2)	81 (53.3)		72 (59.5)	60 (52.2)	
2	53 (22.1)	20 (24.1)	32 (21.1)		24 (19.8)	28 (24.4)	
3–4	34 (14.2)	11 (13.3)	22 (14.5)		17 (14.1)	17 (14.8)	
Number of palliative care visits				0.09			0.20
0	26 (10.1)	11 (12.4)	15 (9.1)		12 (9.2)	14 (11.3)	
1	62 (24.0)	23 (25.8)	38 (23.2)		30 (23.1)	31 (25.0)	
2–3	74 (28.7)	29 (32.6)	41 (25.0)		33 (25.4)	39 (31.5)	
≥4	96 (37.2)	26 (29.2)	70 (42.7)		55 (42.3)	40 (32.3)	

*Note*: Five participants did not have a rating for the “visited family physician for palliative care in last 6 months” outcome, and four did not have a rating for the “scheduled/plan to schedule appointment with family physician” outcome. Additional missing characteristic data for “visited family physician for palliative care in last 6 months” outcome: for marital status, 1 “yes” and 1 “no” participant; for ESASr‐CS total, 3 “yes” and 9 “no” participants; for ESASr‐CS physical, 3 “yes” and 9 “no” participants; for ESASr‐CS emotional, 5 “yes” and 10 “no” participants; and for PRFS, 6 “yes” and 12 “no” participants. Additional missing characteristic data for “scheduled/plan to schedule appointment with family physician” outcome: for marital status, 1 “yes” and 1 “no” participant; ESASr‐CS total, 6 “yes” and 7 “no” participants; for ESASr‐CS physical, 6 “yes” and 7 “no” participants; for ESASr‐CS emotional, 8 “yes” and 8 “no” participants; and for PRFS, 9 “yes” and 9 “no” participants.

Abbreviations: ESASr‐CS, Edmonton Symptom Assessment System‐revised plus constipation and sleep; PRFS, Patient‐Reported Functional Status rating; SD, standard deviation.

Table 2 summarizes enabling factors for patients' visits to the FP. More than 60% of patients indicated that they had been with their FP for more than 5 years, and almost half for more than 10 years. Few patients indicated that their FP offered home visits during (23/256, 9.0%) or after office hours (21/255, 8.2%) or 24‐h telephone support services (54/255, 21.2%). The majority (147/246, 59.8%) disagreed or strongly disagreed that it was easy to reach their FP or the FP's team after hours.

**TABLE 2:** Enabling factors for visits to family physician

Characteristics	Total (*N* = 258), *n* (%)
Length of time with family physician	
<1 year	30 (11.7)
1–5 years	65 (25.3)
6–10 years	42 (16.3)
>10 years	120 (46.7)
Travel time to family physician	
<10 min	75 (29.2)
11–30 min	112 (43.6)
31–60 min	58 (22.6)
>1 h	12 (4.7)
Home visits during office hours	
Yes	23 (9.0)
No/do not know	233 (91.0)
Home visits after office hours	
Yes	21 (8.2)
No/do not know	234 (91.8)
24‐h telephone support service	
Yes	54 (21.2)
No/do not know	201 (78.8)
Easy to reach family physician's team after hours	
0 (strongly disagree)	70 (28.5)
1	77 (31.3)
2	43 (17.5)
3	30 (12.2)
4 (strongly agree)	26 (10.6)

*Note*: Missing data: for length of time with family physical and for travel time, 1 participant; for home visits during office hours, 2 participants; for home visits after office hours, 3 participants; for 24‐h telephone support, 3 participants; and for “easy to reach family physician,” 12 participants.

### 3.2 Current and preferred providers of medical care

Table 3 identifies the different clinicians currently involved in provision of non‐cancer and cancer‐related medical care and patients' preferences for providers of such care. FPs were the main providers of non‐cancer related acute care (193/254, 76.0%) and non‐cancer chronic medical management (176/254, 69.3%) and were also the preferred healthcare providers for both types of care (218/252, 86.5%, and 221/253, 87.4%, respectively). In contrast, few FPs were involved in cancer‐related care, including coordination of cancer care and pain and symptom management for cancer and cancer treatments (0.8%–2.0%), nor were they the preferred providers for such care (2.0%–4.1%). Rather, palliative care clinicians tended to be the actual and preferred providers of cancer‐related pain and symptom management, whereas oncologists were most commonly the actual and preferred providers of care for cancer treatment‐related symptoms. Few patients indicated that FPs provided psychosocial palliative care services, including emotional care, advance care directives, support for caregivers and family members, and arrangement of home‐care services (2.8%–6.0%), although a somewhat larger proportion of patients preferred FPs to provide such care (12.6%–27.6%). Compared to FPs and oncologists, the largest proportions of patients reported that palliative care clinicians were their actual (10.7%–29.6%) and preferred (57.8%–68.9%) providers of psychosocial care.

**TABLE 3:** Current and preferred providers for different types of medical care

	Medical care providers, *n* (%)				
Type of medical care	Family doctor	Palliative care team	Oncologist/oncology team	Other healthcare provider^a^	Do not receive this care^a^
Non‐cancer related care (e.g., common cold, flu)					
Receive	193 (76.0)	2 (0.8)	9 (3.5)	10 (3.9)	40 (15.7)
Prefer to receive	218 (86.5)	18 (7.1)	16 (6.3)	—	—
Non‐cancer chronic medical management (e.g., high blood pressure)					
Receive	176 (69.3)	3 (1.2)	8 (3.1)	20 (7.9)	47 (18.5)
Prefer to receive	221 (87.4)	19 (7.5)	13 (5.1)	—	—
Coordination of cancer care					
Receive	5 (2.0)	34 (13.9)	193 (79.1)	4 (1.6)	8 (3.3)
Prefer to receive	10 (4.1)	54 (22.2)	179 (73.7)	—	—
Pain management for cancer					
Receive	3 (1.2)	197 (78.5)	25 (10.0)	1 (0.4)	25 (10.0)
Prefer to receive	5 (2.0)	213 (84.9)	33 (13.1)	—	—
Symptom management related to treatment for cancer (e.g., nausea from chemotherapy or radiation)					
Receive	2 (0.8)	71 (29.0)	140 (57.1)	5 (2.0)	27 (11.0)
Prefer to receive	5 (2.0)	107 (43.3)	135 (54.7)	—	—
Symptom management for cancer (e.g., nausea, constipation, shortness of breath)					
Receive	5 (2.0)	111 (44.6)	106 (42.6)	6 (2.4)	21 (8.4)
Prefer to receive	6 (2.5)	130 (53.3)	108 (44.3)	—	—
Emotional care related to my cancer (e.g., for anxiety, sadness)					
Receive	15 (6.0)	41 (16.4)	9 (3.6)	61 (24.4)	124 (49.6)
Prefer to receive	64 (27.6)	134 (57.8)	34 (14.7)	—	—
Advance care directives support (planning for the future in case you are no longer able to make decisions for yourself)					
Receive	8 (3.2)	74 (29.6)	4 (1.6)	15 (6.0)	149 (59.6)
Prefer to receive	41 (17.2)	164 (68.9)	33 (13.9)	—	—
Providing support for your caregiver/family					
Receive	10 (4.0)	27 (10.7)	7 (2.8)	19 (7.5)	189 (75.0)
Prefer to receive	50 (21.8)	145 (63.3)	34 (14.8)	—	—
Arranging home care and related services (e.g., community care access center)					
Receive	7 (2.8)	49 (19.8)	46 (18.6)	30 (12.1)	115 (46.6)
Prefer to receive	30 (12.6)	154 (64.7)	54 (22.7)	—	—

^a^ Response options provided only for “Receive.” Number of participants who did not indicate a medical care provider: for non‐cancer related care, 4 “receive” and 6 “prefer” participants; for non‐cancer medical management, 4 “receive” and 5 “prefer” participants; for coordination of cancer care, 14 “receive” and 15 “prefer” participants; for pain management for cancer, 7 “receive” and 7 “prefer” participants; for symptom management (cancer treatment), 13 “receive” and 11 “prefer” participants; for symptom management (cancer symptoms), 9 “receive” and 14 “prefer” participants; for emotional care, 8 “receive” and 26 “prefer” participants; for advance care directions, 8 “receive” and 20 “prefer” participants; for caregiver/family support, 6 “receive” and 29 “prefer” participants; and for home care arrangements, 11 “receive” and 20 “prefer” participants.

### 3.3 Perception of FP's medical care by previous and planned visits to the FP

Table 4 summarizes the number of participants who agreed or strongly agreed with statements of positive perceptions of care received from their FP. Patients were most likely to endorse the item “knows me as a person” and least likely to endorse the item “able to provide the time I need to address all of my problems.” Almost all statements were more likely to be endorsed by those who had visited their FP for palliative care in the past 6 months than by those who had not. Feeling that the FP provided sufficient time to address problems was the only statement that was significantly more likely to be endorsed by those who had a scheduled or planned appointment with their FP than by those who did not (OR = 1.29, 95% CI = 1.07–1.55, *p* = 0.01). The total score, reflecting positive perceptions of overall medical care, was significantly associated with both having visited the FP for palliative care in the last 6 months (OR = 1.06, 95% CI = 1.02–1.11, *p* = 0.002) and having a scheduled or planned appointment with the FP (OR = 1.03, 95% CI = 1.00–1.07, *p* = 0.049).

**TABLE 4:** Perceptions of medical care by previous or planned visits to family physicians

Perceptions of medical care	Visited family physician for palliative care in last 6 months, *n* (%) agree/strongly agree					Scheduled/plan to schedule appointment with family physician, *n* (%) agree/strongly agree				
	Obs N	Yes	No	OR (95% CI)	*p*	Obs N	Yes	No	OR (95% CI)	*p*
I am satisfied with the care I get from my Family Doctor	248	68 (78.2)	100 (62.1)	1.37 (1.10–1.69)	0.004	250	93 (72.7)	77 (63.1)	1.13 (0.94–1.35)	0.20
I am confident that I am receiving the best possible care from my Family Doctor	239	57 (67.1)	89 (57.8)	1.25 (1.03–1.51)	0.03	241	79 (63.2)	68 (58.6)	1.18 (0.99–1.41)	0.06
My Family Doctor's role in my healthcare is clear	249	66 (74.2)	90 (56.3)	1.46 (1.16–1.82)	0.001	250	84 (66.1)	73 (59.3)	1.17 (0.96–1.42)	0.11
My Family Doctor is able to provide the time I need to address all of my problems	249	60 (67.4)	80 (50.0)	1.42 (1.15–1.73)	0.001	250	81 (62.8)	60 (49.6)	1.29 (1.07–1.55)	0.01
My Family Doctor knows me as a person	250	72 (82.8)	119 (73.0)	1.35 (1.05–1.74)	0.02	251	103 (79.8)	89 (73.0)	1.21 (0.97–1.50)	0.08
My Family Doctor provides me with prompt care	250	65 (73.0)	103 (64.0)	1.19 (0.96–1.49)	0.11	252	91 (71.1)	79 (63.7)	1.14 (0.93–1.40)	0.20
My Family Doctor is available for the services I want him/her to provide	253	66 (74.2)	99 (60.4)	1.32 (1.05–1.66)	0.02	254	82 (63.1)	85 (68.5)	1.14 (0.93–1.40)	0.22
Satisfaction with care total score, mean (SD)	253	27.8 (6.8)	24.6 (7.6)	1.06 (1.02–1.11)	0.002	254	26.6 (7.5)	24.8 (7.5)	1.03 (1.00–1.07)	0.049

*Note*: OR indicates per‐level increase in agreement. *P*‐values are derived from univariable binary logistic regression analyses that regressed each item on each outcome. The seven perception items were combined into a single score reflecting perceptions of the family physician's medical care. Five participants did not have a rating for the “visited family physician for palliative care in last 6 months” outcome, and four did not have a rating for the “scheduled/plan to schedule appointment with family physician” outcome.

Abbreviations: CI, confidence intervals; Obs N, observed sample sizes in descriptive analyses; OR, odds ratios; SD, standard deviation.

### 3.4 Multivariable factors associated with previous and planned visits to the FP

Results of the multivariable analyses are shown in Table 5. Three FP factors remained negatively or positively associated with having visited the FP for palliative care in the past 6 months: travel time to the FP (OR = 0.67, 95% CI = 0.48–0.93, *p* = 0.02), indicating that the FP offers 24‐h telephone support (OR = 1.96, 95% CI = 1.02–3.76, *p* = 0.04), and better perceived care by the FP (OR = 1.05, 95% CI = 1.01–1.09, *p* = 0.01).

**TABLE 5:** Multivariable factors associated with previous and planned visits to family physician

Factors	Visited family physician for palliative care in last 6 months			Scheduled/plan to schedule appointment with family physician		
	OR	95% CI	*p*	OR	95% CI	*p*
Patient sex, female	—	—	—	0.51	0.30–0.87	0.01
First language, English	—	—	—	2.90	1.04–8.11	0.04
Travel time to family physician	0.67	0.48–0.93	0.02	0.66	0.47–0.93	0.02
24‐h telephone support service, yes	1.96	1.02–3.76	0.04	—	—	—
Easy to reach family physician after hours	—	—	—	1.33	1.08–1.64	0.008
Perception of medical care, total score	1.05	1.01–1.09	0.01	—	—	—

*Note*: Factors with *p* < 0.05 were retained in the multivariable model. Univariable factors meeting the *p* < 0.25 selection criterion for inclusion in the multivariable analyses for “visited family physician for palliative care in last 6 months” were: travel time to family physician (OR = 0.66 [95% CI = 0.48–0.92]), 24‐h telephone support service (OR = 2.43 [95% CI = 1.30–4.52]), number of palliative care visits (OR = 0.80 [95% CI = 0.62–1.04]), “easy to reach family physician after hours” (OR = 1.27 [95% CI = 1.04–1.56]), and perception of medical care (OR = 1.06 [95% CI = 1.02–1.11]). Univariable factors meeting the *p* < 0.25 criterion for inclusion in the multivariable analyses for “scheduled/plan to schedule appointment with family physician” were: sex (female) (OR = 0.54 [95% CI = 0.33–0.89]), European ethnicity (OR = 1.67 [95% CI = 0.98–2.87]), first language English (OR = 3.06 [95% CI = 1.16–8.10]), travel time to family physician (OR = 0.68 [95% CI = 0.50–0.92]), home visits after office hours (OR = 2.05 [95% CI = 0.80–5.27]), 24‐h telephone support service (OR = 1.77 [95% CI = 0.95–3.29]), number of palliative care visits (OR = 1.17 [95% CI = 0.92–1.50]), “easy to reach family physician after hours” (OR = 1.40 [95% CI = 1.14–1.71]), and perception of medical care (OR = 1.03 [95% CI = 1.00–1.07]).

Abbreviations: CI, confidence intervals; OR, odds ratios.

The following variables were negatively or positively associated with having a scheduled appointment with the FP: female sex (OR = 0.51, 95% CI = 0.30–0.87, *p* = 0.01), English as first language (OR = 2.90, 95% CI = 1.04–8.11, *p* = 0.04), travel time to the FP (OR = 0.66, 95% CI = 0.47–0.93, *p* = 0.02), and ease of reaching the FP after hours (OR = 1.33, 95% CI = 1.08–1.64, *p* = 0.008).

## 4 DISCUSSION

In our study, approximately one‐third of patients had seen their FP for palliative care in the last 6 months, and half had a scheduled or planned FP's appointment. Enabling factors associated with having visited FPs for palliative care or with having a scheduled or planned appointment included shorter travel time to FPs, 24‐h telephone support services, and ease of reaching FPs after hours. In addition, a positive perception of the FP's care was associated with having seen the FP for palliative care in the last 6 months. Female patient sex was negatively associated, and English as a first language was positively associated, with having scheduled or planning to schedule a FP visit. The number of previous visits to the palliative care clinic was not associated with either outcome.

The majority of participants indicated that their FPs knew them well and expressed general satisfaction with the FP's care. However, there was less endorsement of adequate time to address problems and of confidence in receiving the best care possible. Moreover, few patients indicated that FPs provided palliative care services such as cancer‐related symptom management, emotional care, discussions of advance care directives, support for caregivers and family, and provision or arrangement of home care.^8^, ^9^, ^10^, ^11^, ^12^ FPs have reported barriers to providing palliative care, including insufficient time^22^, ^24^; lack of resources including education and training in palliative care^22^, ^24^; poor integration and communication with other healthcare providers; and ambiguity about their role in end‐of‐life care.^13^, ^20^, ^21^, ^22^, ^23^, ^24^ Given that FPs' involvement in palliative care may enable holistic care, decrease emergency room visits, and increase the likelihood of dying at home,^13^, ^32^ additional supports and resources are needed to enable this involvement. These could include training and education, better integration and communication with hospital‐based services, and compensation for care provision.^5^, ^13^, ^22^

Enabling factors, including shorter travel time to the FP's office, availability of 24‐h telephone support, and after‐hours services, were associated with previous visits to the FP for palliative care and/or with planned FP visits. Ease of access to FPs is particularly important for patients with advanced cancer, for whom fatigue is a prominent symptom^33^, ^34^, ^35^ and whose condition may worsen unexpectedly. FPs' provision of after‐hours care was similarly deemed important in a survey of patients attending a radiation oncology clinic.^36^ Group practices, out‐of‐hours cooperatives, and better remuneration for after‐hours care may facilitate FPs' provision of 24‐h care.^13^, ^37^

Additional factors were associated with both outcomes. Female patients were less likely to have a planned appointment with FPs, which suggests that they were more likely to seek care exclusively from the palliative care team. Indeed, previous research demonstrated that female patients are more likely to know about and receive palliative care.^38^, ^39^ English as a first language was also positively associated with having a scheduled or planned FP appointment. Canadian patients have reported lower rates of same‐day response from their family physicians if their first language is neither English nor French (the official Canadian languages).^40^ Extending telephone translation services that are available in many hospitals (including Princess Margaret Cancer Centre) to primary care settings would promote equitable access to primary care. Lastly, having seen FPs for palliative care services in the last 6 months was associated with a positive perception of FPs' care, with sufficient time to address patients' problems and clarity of the FP's role being deemed particularly important. Although providing sufficient time for consultations may be challenging in a busy family practice,^37^ patients with advanced cancer value patient‐led, unhurried palliative care visits.^15^, ^41^ Role clarity of the FP in this regard may be increased by patient education to explain the relevance and value of their FP as an active provider of palliative care.

The observed importance of enabling and predisposing factors is relevant for models of integration of primary care into models of comprehensive, coordinated palliative care.^4^, ^5^, ^6^, ^7^ Patients with advanced disease desire continuous care that is accessible and close to home, and FPs are well placed to meet such needs.^13^ Integrated care pathways and ways to redress challenges FPs face in providing palliative care have been advanced^13^ but must be enacted concurrently with patient education about the role of FPs in palliative care.

A limitation of this study is that all participants were attending an outpatient palliative care clinic and that only 10% of participants were attending the clinic for the first time. Although the number of previous visits to the palliative care clinic was not associated with either outcome in the regression analyses, comparative studies with patients who have not been referred to a specialty palliative care clinic care would be informative. However, that most patients were acquainted with a specialty palliative care service was also a strength, as their understanding of palliative care may have helped them to determine whether they would like to receive these services from their FP. Although patients had advanced disease and were receiving palliative care, they reported relatively low symptom burden; findings may not generalize to patients with worse symptom severity. Generalizability may also be limited due to the inclusion criterion of English fluency.

In conclusion, the present study identified enabling factors reflecting ease of access to FPs, predisposing factors of sex and English as the first language, and positive perceptions of FPs' care as correlates of having received palliative care services from FPs or having a planned or scheduled FP appointment. Further research should investigate interventions to promote FP‐provided palliative care.

## AUTHOR CONTRIBUTIONS

**Christine C. Moon:** Conceptualization (equal); data curation (lead); investigation (equal); methodology (equal); writing – original draft (equal); writing – review and editing (equal). **Kenneth Mah:** Formal analysis (equal); writing – original draft (equal); writing – review and editing (equal). **Ashley Pope:** Data curation (supporting); project administration (lead); writing – review and editing (equal). **Nadia Swami:** Investigation (equal); project administration (supporting); writing – review and editing (equal). **Breffni Hannon:** Investigation (equal); writing – review and editing (equal). **Jenny Lau:** Investigation (equal); writing – review and editing (equal). **Ernie Mak:** Investigation (equal); writing – review and editing (equal). **Ahmed Al‐Awamer:** Investigation (equal); writing – review and editing (equal). **Subrata Banerjee:** Investigation (equal); writing – review and editing (equal). **Laura A. Dawson:** Investigation (equal); methodology (equal); writing – review and editing (equal). **Amna Husain:** Investigation (equal); methodology (equal); writing – review and editing (equal). **Gary Rodin:** Investigation (equal); methodology (equal); writing – review and editing (equal). **Lisa W. Le:** Conceptualization (equal); formal analysis (equal); methodology (equal); writing – review and editing (equal). **Camilla Zimmermann:** Conceptualization (equal); investigation (equal); methodology (equal); writing ‐ original draft (equal); writing ‐ review and editing (equal).

## FUNDING INFORMATION

This study was funded by the Canadian Institutes of Health Research (Grant No. 152996 to Dr. Zimmermann). The funding body had no role in the design of the study, collection, analysis, and interpretation of data or in writing the manuscript.

## CONFLICT OF INTEREST

We declare that we have no competing conflicts of interest.

## ETHICS APPROVAL STATEMENT

This study was conducted in line with the principles of the Declaration of Helsinki. Approval was granted by the University Health Network Research Ethics Board (REB #16‐5061‐CE).

## Data Availability

The data that support the findings of this study are available from the corresponding author upon reasonable request.
